# Efficacy of Recombinant Human BMP2 and PDGF-BB in Orofacial Bone Regeneration: A Systematic Review and Meta-analysis

**DOI:** 10.1038/s41598-019-44368-z

**Published:** 2019-05-30

**Authors:** Feifei Li, Fanyuan Yu, Xueyang Liao, Chenzhou Wu, Yitian Wang, Chunjie Li, Feng Lou, Boer Li, Bei Yin, Chenglin Wang, Ling Ye

**Affiliations:** 1 State Key Laboratory of Oral Diseases & National Clinical Research Center for Oral Diseases, West China Hospital of Stomatology, Sichuan University, Chengdu, China; 2 Department of Endodontics, West China Stomatology Hospital, Sichuan University, Chengdu, China; 3 Department of Pediatric Dentistry, West China Hospital of Stomatology, Sichuan University, Chengdu, China; 4 Department of Oral Maxillofacial Surgery, West China Hospital of Stomatology, Sichuan University, Chengdu, China

**Keywords:** Outcomes research, Translational research

## Abstract

With the rapid development of tissue engineering therapies, there is a growing interest in the application of recombinant human growth factors (rhGFs) to regenerate human orofacial bones. However, despite reports of their ability to promote orofacial bone regeneration in animal experiments, their benefits in human clinical treatments remain unclear. Furthermore, the appropriate concentrations or indications of a specific rhGF remain ambiguous. Therefore it is essential to collect data from diverse clinical trials to evaluate their effects more precisely. Here we reviewed randomized clinical trials (RCT) that focused on the utilization of rhGFs in orofacial bone regeneration. Data from included studies were extracted, pooled and then quantitatively analyzed according to a pre-established protocol. Our results indicate that all current concentrations of rhBMP-2 produces insufficient effect on promoting either tooth extraction socket healing, sinus augmentation or reconstruction of alveolar clefts. However, 0.3 mg/ml rhPDGF-BB promotes the healing of tooth extraction sockets, though the effect does not reach a level of statistical significance. Summarily, we recommend concentrations of 0.3 mg/ml rhPDGF-BB only for the healing of tooth extraction sockets.

## Introduction

Numerous patients around the world suffer from orofacial bone loss due to traumas, inflammation, developmental abnormalities and other diseases^1^. Considering the essential functions of orofacial bones, such as preservation of masticatory function, speaking ability and facial esthetics, the loss of these bones has serious negative effects on the quality of life of these patients^1^. The facilitation of bone regeneration therefore constitutes a major concern and challenge for orofacial surgeons^2,3^. Local application of rhGFs emerged as a promising therapeutic strategy^4^. Certain GFs, especially bone morphogenetic protein 2 (BMP-2), has shown great osteotropic potential in animal experiments^5,6^. In addition, orofacial surgery provides an ideal context for the utilization of rhGFs, which can be loaded in scaffold carriers and administrated to block areas of orofacial bones^5,6^.

Several other substances besides rhBMP-2 have often been used in clinical treatments of orofacial bone. These include recombinant human bone morphogenetic protein 7 (rhBMP-7), recombinant human platelet derived growth factor BB (rhPDGF-BB), recombinant human fibroblast growth factor 2 (rhFGF-2) and recombinant human growth differentiation factor 5 (rhGDF-5)^5,6^. These have been used in a variety of therapeutic contexts, including alveolar reconstruction, sinus augmentation, tooth extraction socket healing, implant guided bone regeneration, and periodontal bone repair^5^. However, despite a plethora of studies carried out on animals and humans, a lack of consensus continues to exist concerning the clinical efficacy of rhGFs in different fields of orofacial bone regeneration^6^. Our systematic review of related studies has permitted identification of factors generating the confusion. In the first place, the conclusions have been inconsistent. This has occurred not only in RCTs but also in previous evidence-based studies and meta-analyses. For instance, in 1997, Boyne *et al*. were among the first to indicate the potential clinical efficacy of rhBMP-2 in human sinus augmentation^7^. However, Kao *et al*. in 2012 showed the opposite^8^. They reported significant impairment, both histological and histomorphometrical, of bone regeneration on use of rhBMP-2 in clinical treatments^8^. Secondly, current researches have failed to determine the most effective concentration of a certain rhGF^6^. Such ambiguity seriously impedes its clinical utilization. Again, using rhBMP-2 as an example, it has been utilized in sinus augmentation, cleft alveolar reconstruction, and implant-guided bone regeneration in different doses^5^. So far, however, clinicians are still unclear about the suitable indications and concentration for rhBMP-2^9,10^. They remain hesitant regarding decisions as to the application of rhGFs in orofacial regeneration.

This has prompted us to undertake a rigorous review of relevant studies to address this issue of differential clinical efficacy. Our intent in undertaking this research was to provide orofacial surgeons quantifiable evidence-based guidelines for the appropriate utilization of rhGFs. Though several previous studies on this matter had already been carried out, we updated the corpus of studies to be analyzed, significantly enlarged the overall sample size, and broadened the evaluation indices beyond those used in previous studies. Moreover, we developed rigorous inclusion and exclusion criteria in order to ascertain more precisely the clinical efficacy of a specific rhGFs for a specific therapeutic indication. This entailed examination of the effectiveness of specific rhGFs when utilized in specific orofacial bone surgeries. Furthermore, we tried to ascertain the most effective concentrations of a given rhGF for orofacial regeneration.

To achieve this we have undertaken a comprehensive search and systematic review of the relevant literature. In doing so we explicitly excluded studies on periodontal repair. Periodontal regeneration involves not only periodontal bone regeneration but also periodontal soft tissue regeneration. This gives rise to several potentially confounding variables, such as different stages of a disease, different levels of inflammation and bacteria control, and others. For this reason, we carried out and published a separate independent survey of the literature on periodontal repair^11^. For the present study we established inclusion and exclusion criteria that targeted RCT. Data were extracted and pooled for purposes of quantitative analysis.

## Methods

This study was conducted according to a standardized protocol; the Preferred Reporting Items for Systematic Reviews and Meta-Analyses (PRISMA). We also utilized the Cochrane Handbook for Systematic Reviews of Interventions^12,13^. We required that all of rhGFs to be analyzed in this study be among those approved for application in clinical treatments.

### Inclusion criteria

#### Types of studies

RCTs, including parallel RCTs and split mouth RCTs, were considered for inclusion. But controlled clinical trials (CCTs), quasi-RCTs, cohort studies, case reports and other studies that fall outside of the category RCT were excluded. To be included in our study, an RCT had to have been approved and guided by a recognized ethics committee.

#### Types of participants

To be considered as a valid participant, patients had to be suffering from orofacial bone diseases or loss, including Cleft lip and palate (CLP), tooth extraction or other traumas that required regenerative treatments. We also included patients in need of orofacial bone reconstruction as a prerequisite for further treatments, such as sinus augmentation before implant surgery or prosthetic treatment. We excluded the studies whose patients received rhGFs in order to repair periodontal bones.

#### Types of interventions

Patients receiving conventional treatments without rhGFs were regarded as the control group. Their clinical outcomes were compared with those of patients receiving rhGFs.

#### Types of outcome measures

Since the analysis was designed to measure the effect of rhGFs in orofacial bone regeneration, we took into account relevant clinical diameters, radiographic markers and histomorphological evaluations.

#### Incidence of other complications

All reports of adverse effects or side effects were excluded from this study.

### Exclusion criteria

Published clinical trials were excluded if they did not meet the above criteria.

### Search methods

The search was restricted to articles written in English. A literature search was carried out within the Cochrane Central Register of Controlled Trials (CENTRAL; 2017), MEDLINE (via OVID, 1948 to December 2017), Embase (1984 to December 2017), the China National Knowledge Infrastructure (CNKI; 1979 to December 2017), the China Biology Medicine disc (CBM; 1978 to December 2017) and Google Scholar. The online databases of related journals were also searched. References listed in published articles were also checked. In order to find ongoing clinical trials, we also searched the World Health Organization International Clinical Trials Registry Platform. The search strategy entailed combination of both MeSH heading words and free key text words.

### Study inclusion

Three reviewers (FYY, FFL and CJL) independently screened and evaluated the titles and abstracts of potential articles utilizing the above-mentioned selection criteria. Then full-texts were further assessed for all studies that possibly met the inclusion criteria or for cases in which it was difficult to make a final decision because of insufficient information. When disagreements came up, they were resolved by consensus. If no consensus was reached, an alternative investigator (LY or CLW) acted as an arbiter. The study selection was shown in supplemental data (Fig. S1).

### Assessment of risk of bias

The Cochrane “risk of bias” instrument was used^13^. Bias evaluation was performed by 4 independent reviewers (BEL, XYL, BY and FL). Disagreements were resolved by discussion until consensus was reached. The risk of bias was classified into three categories:

- Low risk of bias if all domains were marked as “low risk”;
- Moderate risk of bias if no domain was marked as “high risk” but at least one was coded as “unclear risk”;
- High risk of bias if more than one domain was marked as “high risk”.

### Data extraction

The following data were extracted from each of the articles chosen for evaluation: demographic data, method of randomization, randomization concealment and blinding, and measurement outcomes. Two estimators independently extracted data from the included studies (FYY and FFL) using a custom-designed form.

### Statistical analysis

Statistical analysis were carried out utilizing Review Manager 5.1. Heterogeneity was assessed via the I^2^ statistic (a test for heterogeneity) on the level of α = 0.10. If there was considerable or substantial heterogeneity (I^2^ > 50%), a random-effects model was adopted; otherwise a fixed-effects model was used. We handled data of dependent studies in accordance to the guidelines of Cochrane Cochrane Handbook for Systematic Reviews of Intervention^12,13^. The results of treatment effect were presented as median difference (MD) utilizing 95% confidence intervals (CIs). Statistical significance was calculated at α = 0.05 (2-tailed z tests).

## Results

### Search results

Having met the inclusion criteria, the RCTs were analyzed via quantitative meta-analysis (S1). The characteristics of included RCTs are shown in Table 1. The assessments of bias were presented in the forest plot graphs.

**Table 1:** Characteristics of included RCTs.

Study ID	Study type (Study Design)	Patient				Arms					F/U Period (monthes)	Outcomes
		Number	Gender (F/M)	Age	Indications	Intervention			Control			
						growth factor(mg/ml)	Number	Carrier	Item	Number		
Fiorellini 2005	RCT, parallel	80	37/43	47.4	extraction socket healing	0.75 rhBMP-2	21	ACS	ACS	18	4	bone height;bone width;bone volume;Bone density
						1.5 rhBMP-2	20		No carrier	20		
Kim 2014	RCT, parallel	69	35/34	*51.18 ± 10.14 50.37 ± 13.45	extraction socket healing	0.05 rhBMP-2	35	DBM	DBM	34	3	bone height;bone width
Huh 2011	RCT, parallel	72	30/42	*52.75 ± 6.29 52.80 ± 7.20	extraction socket healing	1.5 rhBMP-2	32	TCP/HA	TCP/HA	32	3	bone height;bone width
Coomes 2014	RCT,parallel	39	17/21	*33 to 79 19 to 74	extraction socket healing	1.5 rhBMP-2	20	ACS	ACS	19	5	bone height;bone width
Kim 2015	RCT,parallel	127	34/93	*53.91 ± 6.81 53.15 ± 6.77	sinus augmentation	1 rhBMP-2	65	HA	ABX (Bio-Oss))	62	3	New bone%
Triplett 2009	RCT, parallel	160	89/71	*51.4 53.6	sinus augmentation	1.5 rhBMP-2	82	ACS	autograft	78	6	bone height;bone density
Kao 2012	RCT,parallel	22	9/13	*51.09 50.45	sinus augmentation	1.5 rhBMP-2	11	ACS + Bio-Oss	Bio-Oss	11	6	New bone%
Froum 2014	RCT,split-mouth design	18	—	—	sinus augmentation	1.5 rhBMP-2 (5.6 ml) 1.5 rhBMP-2 (2.8 ml)	12 12	ACS + MCBA	MCBA	12	6–9	bone height;bone density;bone volume
Philip 2005	RCT, parallel	48	29/19	*57 ± 11 57 ± 12 52 ± 9	sinus augmentation	0.75 rhBMP-2 1.5 rhBMP-2	18 17	ACS	bone graft	13	4	bone height;bone width;bone density
Canan Jr 2012	RCT,parallel	18	6/12	*10.8 ± 2.3 8.7 ± 0.5	alveolar reconstruction in CLP	1.5 rhBMP-2	6	ACS	bone graft	6	3-12	bone formation;bone hight;bone density
Alonso 2010	RCT, parallel	16	7/9	9.6	alveolar reconstruction in CLP	1.5 rhBMP-2	8	ACS	autograft	8	12	bone volume; bone hight
Nevins 2011	RCT, parallel	15	—	—	extraction socket healing	0.3 rhPDGF-BB	4	MCBS	MCBS	4	5	new bone%
Geurs 2014	RCT, parallel	41	29/12	52	extraction socket healing	0.3 rhPDGF-BB	9	β−TCP + FDBA	β−TCP + FDBA	11	2	new bone%

*Age range differed in each group(raw data shown in original paper); −: no information; F/M: female number versus male number; F/U, Follow-up; ACS: asorbable collagen sponge; DBM: demineralized bone matrix; TCP: tricalcium phosphate; HA: hydroxyapatite; Bio-Oss: bovine bone xenograft (Geistlich); ABX: an inorganic bovine bone xenograft (ABX); MCBA: mineralized cancellous bone allograft; MCBS: mineral collagen bone substitute; FDBA: mineralized freeze-dried bone allograft.

### The effect of rhBMP-2 for alveolar reconstruction in CLP patients

CLPs are the most common congenital facial deformities^14^. Effective alveolar reconstruction is an important step in the improvement of the appearance and function of CLP patients. We assessed the effect of rhBMP-2 on this therapeutic process.

Three studies deal with the effect of rhBMP-2 in the alveolar reconstruction of CLP patients’: (Dickinson 2007, Alonso 2010 and Canan Jr 2012)^15–17^. Bone formation rate and increased bone volume were studied in two of the studies but not in Dickinson 2007^15–17^. Dickinson 2007 was therefore excluded in evaluating these two parameter^15^. Data on these outcomes were directly extracted from Canan Jr 2012^17^. Alonso 2010, however, contained no information on changes occurring between the immediate post-surgery period and the follow-up endpoint^16^. These two outcomes were therefore indirectly estimated by statisticians according to a previously established method1^9,16^. Both Canan Jr 2012 and Alonso 2010 applied 1.5 mg/ml rhBMP-2 in the intervention group^16,17^.

#### Bone formation rate

**Figure 1:**
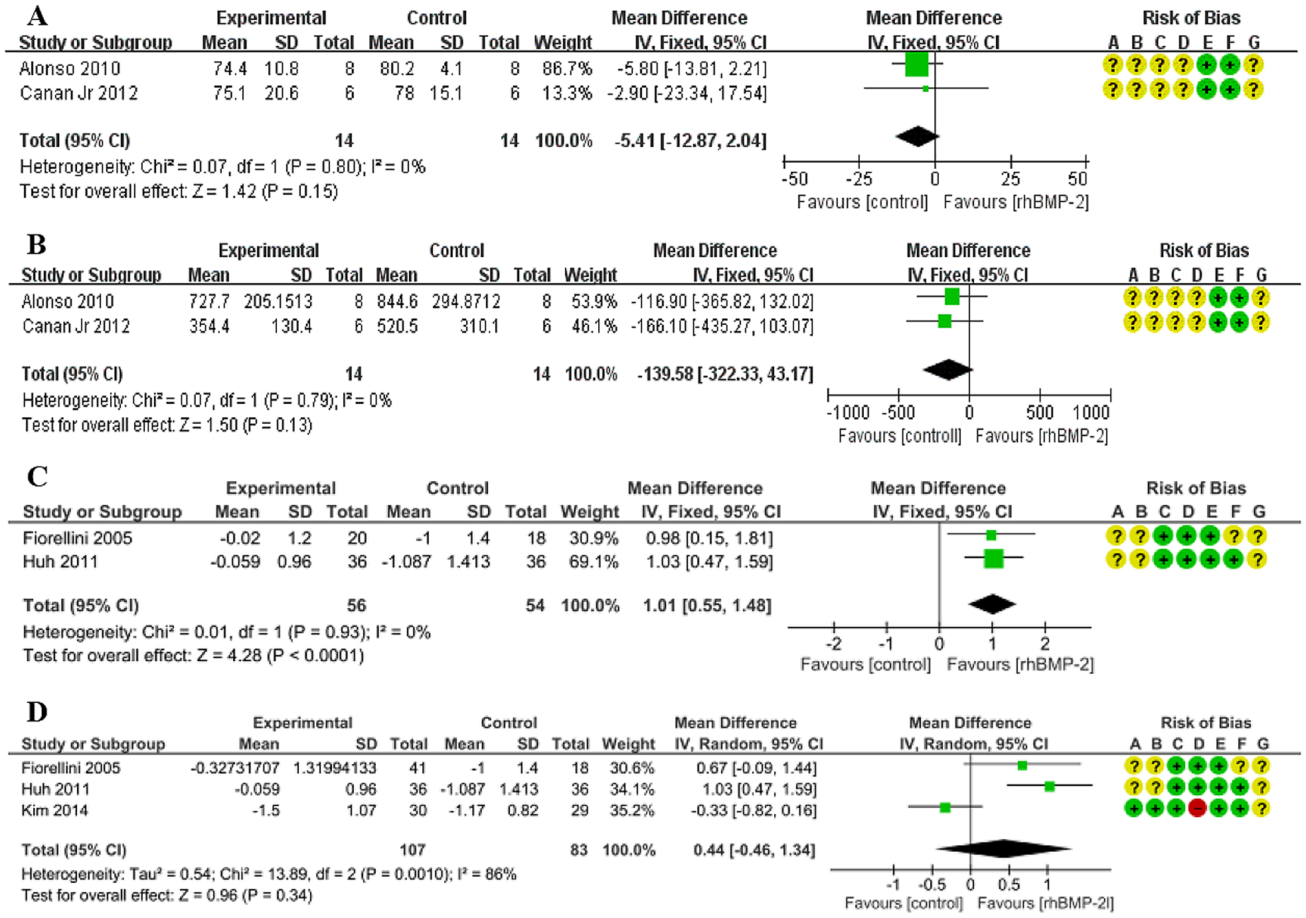
Forest plot of comparison: The effect of rhBMP-2 for alveolar reconstruction in CLP patients and for the healing of tooth extraction socket. (**A**) The measurement of bone formation rate when utilizing a concentration of 1.5 mg/ml rhBMP-2 in CLP patients. (**B**) The measurement of increased bone volume when utilizing a concentration of 1.5 mg/ml rhBMP-2 in CLP patients. (**C**) The measurement of the primary outcome of tooth extraction socket healing, namely alveolar bone height change, when utilizing only a concentration of 1.5 mg/ml rhBMP-2. (**D**) The measurement of the primary outcome of tooth extraction socket healing, namely alveolar bone height change, when taking the results of utilizing three concentrations of rhBMP-2 (0.05 mg/ml, 0.75 mg/ml, 1.5 mg/ml) into consideration. Risk of bias legends: A-random sequence generation (selection bias); B-allocation concealment (selection bias); C-blinding of participants and personnel (performance bias); D-blinding of outcome assessment (detection bias); E-incomplete outcome data (attrition bias); F-selective reporting (reporting bias); G-other bias. +: low risk; ?: unclear risk; −: high risk. All following figures of the risk of bias share the same legends of this figure, therefore this section of legends will not be presented repeatedly in the below figures.

As for the bone formation rate, Alonso 2010 showed that in the rhBMP-2 group the effect was weaker than that found in the control group^16^. In Canan Jr 2012’s study, the mean value of the rhBMP-2 group was 2.9% lower than that of the control group^17^. The overall effect was in favor of the control group (MD = −5.41), though the difference did not reach the 0.05 level of statistical significance (95%CI: −12.87, 2.04; p = 0.15). The heterogeneity of this meta-analysis was low (χ^2^ = 0.07, I^2^ = 0%) (Fig. 1A).

#### Increased bone volume

Increased bone volume is a desired therapeutic outcome in alveolar reconstruction. Alonso 2010 and Canan Jr 2012 measured this outcome on 28 patients^16,17^. These data were extracted and pooled. The control group achieved a higher level (139.58 mm^3^) of increased bone volume than the rhBMP-2 group, though the difference did not reach statistical significance (Corr = 0.5; MD = −139.58; 95% CI: −322.33, 43.17; p = 0.13). The heterogeneity of this meta-analysis was low (χ^2^ = 0.07, I^2^ = 0%) (Fig. 1B).

The above results indicate that the application of 1.5 mg/ml rhBMP-2 during the alveolar reconstruction of CLP patients fails to achieve any positive effect.

### The effect of rhBMP-2 for tooth extraction socket healing

Successful prosthetic and implant treatment following tooth extraction presupposes the restoration of adequate bone volume^18^. The success of socket healing is measured by the level of bone volume restoration. Both alveolar bone height and width in extraction sites are therefore important measures of the effectiveness of treatment.

Five studies reported the effect of the application of rhBMP-2 on alveolar bone regeneration after tooth extraction. After our review of the full text, however, Bianchi 2004 was excluded because its data were retrospectively gathered from one center (20 subjects) of an 80-subject multicenter trial^18,19^. The remaining four RCTs met our inclusion criteria (Coomes 2014, Fiorellini 2005, Kim 2014 and Huh 2011)^20–23^. The primary outcome of alveolar bone healing after tooth extraction is the change in bone height. The secondary outcome is the change in bone width.

#### Alveolar bone height change

Huh 2011 applied a 1.5 mg/ml concentration of rhBMP-2 and Kim 2014 applied 0.05 mg/ml during therapeutic intervention^22,23^. Fiorellini 2005, in contrast, applied two intervention tests. In one of them the concentration of rhBMP-2 was 0.75 mg/ml, whereas the other utilized a concentration of 1.5 mg/ml^21^. To measure the effect of 1.5 mg/ml rhBMP-2, we extracted data from Fiorellini 2005 and Huh 2011^21,23^. Meta-analysis reveals that the mean difference was 1.01 mm in favor of 1.5 mg/ml rhBMP-2, and the difference achieved statistical significance (95%CI:0.55, 1.48; p < 0.0001) (Fig. 1C). Without taking into consideration the different concentration levels, the 0.75 mg/ml rhBMP-2 test in Fiorellini 2005 and the 0.05 mg/ml arm in Kim 2014 were also included^21,22^. Merging the different concentration levels, the combined outcome was 0.44 mm in favor of the rhBMP-2 group, but the difference again failed to reach statistical significance (95% CI: −0.46, 1.34; p = 0.34) (Fig. 1D).

#### Alveolar bone width change

**Figure 2:**
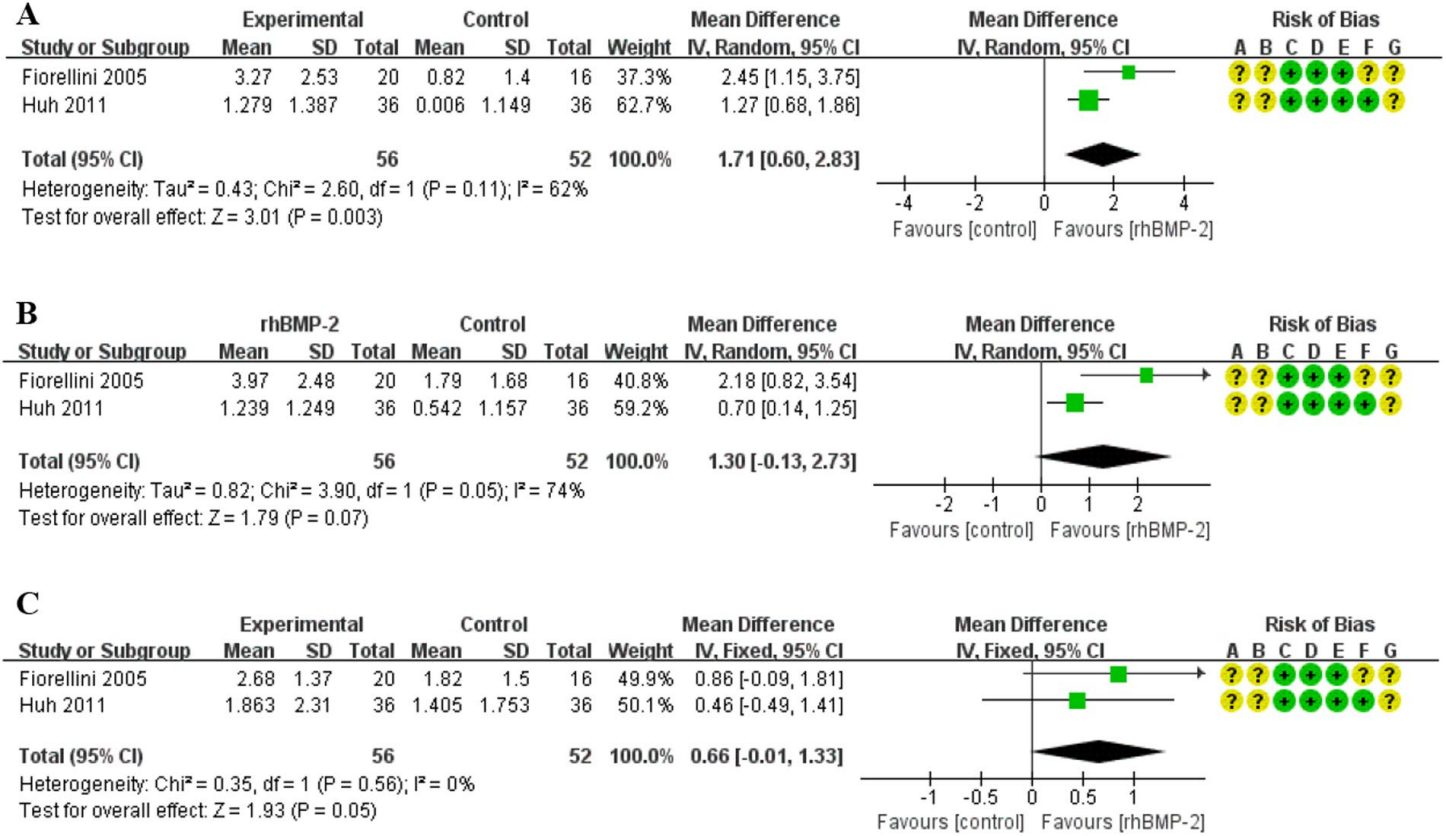
Forest plot of comparison: The effect of only 1.5 mg/ml rhBMP-2 for the healing of tooth extraction socket; outcome: alveolar bone width change. (**A**) The assessment of alveolar bone width change at the 25% distance from alveolar crest to socket bottom. (**B**) The assessment of alveolar bone width change at the 50% distance from alveolar crest to socket bottom. (**C**) The assessment of alveolar bone width change at the 75% distance from alveolar crest to socket bottom.

Bone width change, the secondary outcome in tooth socket’s bone healing, was measured. At the 25% distance from alveolar crest to socket bottom, meta-analysis showed a statistically significant difference between the treatment and control groups. The treatment group had 1.71 mm greater bone width than the control group after treatment with 1.5 mg/ml rhBMP-2. (95%CI: 0.60, 2.83; p = 0.003) (Fig. 2A).

**Figure 3:**
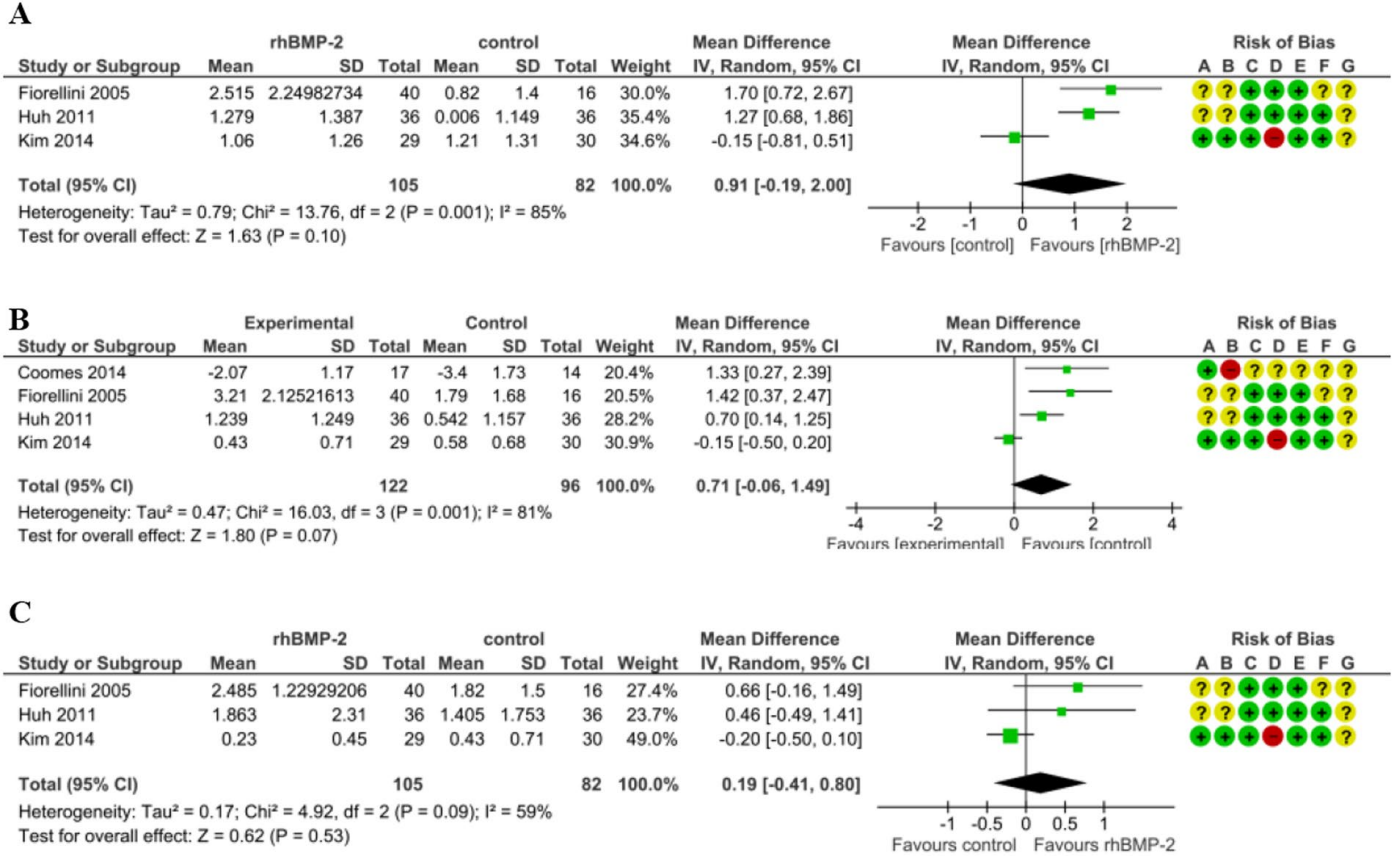
Forest plot of comparison: The effect of three concentration of rhBMP-2 (0.05 mg/ml, 0.75 mg/ml, and 1.5 mg/ml) for the healing of tooth extraction socket; outcome: alveolar bone width change. (**A**) The assessment of alveolar bone width change at the 25% distance from alveolar crest to socket bottom. (**B**) The assessment of alveolar bone width change at the 50% distance from alveolar crest to socket bottom. (**C**) The assessment of alveolar bone width change at the 75% distance from alveolar crest to socket bottom.

Furthermore, at the 50% distance and the 75% distance the overall effect were respectively 1.30 mm and 0.66 mm in favor of the 1.5 mg/ml rhBMP-2 treatment group without statistical significance (Fig. 2B,C). We combined data from studies that used different concentration levels (0.05 mg/ml, 0.75 mg/ml and 1.5 mg/ml rhBMP-2) in the treatment groups. At the 25% distance from the alveolar crest to the socket bottom the treatment group displayed a wider distance (0.91 mm) than the control group. (95%CI: −0.19, 2.00; p = 0.10) (Fig. 3A). Similarly, at the 50% distance the combined effect was in favor of the rhBMP-2 group. (MD = 0.71, 95%CI: −0.06, 1.49; p = 0.07) (Fig. 3B). At the 75% distance from the alveolar crest to the socket bottom the rhBMP-2 groups showed 0.19 mm greater width than controls. Though the difference was in the predicted direction, it was not statistically significant (95%CI: −0.41, 0.80; p = 0.53) (Fig. 3C).

### The effect of rhBMP-2 for sinus augmentation

The success of dental surgeries, prosthetic reconstruction, and implant placement in the anterior maxilla commonly entails the restoration of an adequate level of bone volume. However, there are various confounding factors, such as age-related bone decrease, severe periodontal diseases and other factors, that can have an impact on bone volume. In any case, the maxillary sinus lift procedure has been found to be of great importance in the restoration of an adequate volume to the anterior maxillary bone. The major desired outcomes of sinus augmentation are an increase in the height of the anterior maxillary alveolar bone and an increase in the rate of new bone formation. Secondary outcomes are an increase in alveolar bone width and an increase in the density of new bones.

#### Alveolar bone height change

To measure the change in alveolar height, Froum 2014 and Triplett 2009 applied 1.5 mg/ml rhBMP-2 to the treatment group^24,25^. In Froum 2014, there were two different intervention groups, each of which received the same concentration level (1.5 mg/ml) but applied in different volumes (2.8 ml and 5.6 ml respectively)^24^. Philip 2005 adopted 0.75 mg/ml in one experimental group and 1.5 mg/ml in the other experimental group^26^. In Froum 2014, in the absence of precise information on the changes in bone height, the outcomes were estimated by statisticians based on measures of alveolar height gathered at the baseline phase and the follow-up endpoint^24^.

**Figure 4:**
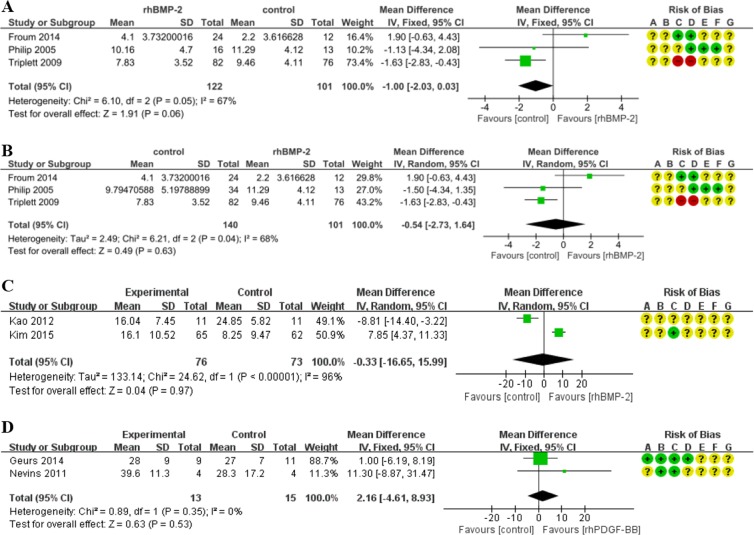
Forest plot of comparison: The effect of rhBMP-2 for sinus augmentation and the effect of rhPDGF-BB for the healing of tooth extraction socket. (**A**) The assessment of 1.5 mg/ml rhBMP-2 for sinus augmentation; outcome: alveolar bone height change. (**B**) The assessment of 0.75 mg/ml and 1.5 mg/ml rhBMP-2 for sinus augmentation; outcome: alveolar bone height change. (**C**) The assessment of 1.5 mg/ml and 1 mg/ml rhBMP-2 for sinus augmentation; outcome: morphometric bone formation rate. (**D**) The effect of 0.3 mg/ml rhPDGF-BB on the healing of tooth extraction socket; outcome: bone formation rate.

We extracted and pooled all data on changes in alveolar bone height during RCTs whose intervention group received 1.5 mg/ml of rhBMP-2. Our meta-analysis revealed that the application of a 1.5 mg/ml concentration level of rhBMP-2 resulted in a less increment of alveolar height (−1.00 mm) than occurred in the control group. The difference is not statistically significant (Corr = 0.5; 95%CI: −2.03, 0.03; p = 0.06). The results are characterized by a high level of heterogeneity (χ^2^ = 6.10, I^2^ = 67%) (Fig. 4A). After including the data of 0.75 mg/ml rhBMP-2 group, the overall effect of meta-analysis also acted in favor of the control. The difference lacked statistical significance (MD = −0.54, 95%CI = −2.73, 1.64, p = 0.63). The heterogeneity of this meta-analysis was high (χ^2^ = 6.21, I^2^ = 68%) (Fig. 4B).

#### Morphometric bone formation rate

Kao 2012 and Kim 2015 measured this outcome^8,27^. The analysis of these data demonstrated that the overall effect was unfavorable for the treatment group, though the differences were not statistically significant. (MD = −0.33; 95%CI: −16.65, 15.99; p = 0.97) (Fig. 4C). The heterogeneity of this study was very high probably resulting from the difference of concentrations between these two studies (Table 1).

To sum up briefly, with respect to the primary outcome of sinus augmentation, rhBMP-2 appears to have no measurable effect on the rate of new bone formation; neither does it give evidence of producing any significant increase in the alveolar height.

#### Secondary outcomes

In our meta-analysis we explored one secondary outcome. With respect to the effect of treatment on increasing bone width, only Philip 2005 evaluated this aspect. Therefore this outcome can’t be quantitatively measured. In terms of the effect of treatment concentration on new bone density, the data indicate that the 1.5 mg/ml rhBMP-2 concentration level tends to have no positive effect (MD = −55.74). Once again, however, the differences were not statistically significant (95%CI: −351.03, 239.55; p = 0.71) (S2-A). When the differences in concentration level were ignored and the results of all concentration levels were combined into one variable, the overall effect continued to be in favor of the control group, again without statistical significance (MD = −71.14; 95%CI: −394.86, 252.57; p = 0.67) (S2-B). Details of secondary outcomes are presented as supplemental data.

In summary, the use of rhBMP-2 fails to improve the secondary outcome of sinus augmentation.

### The effect of rhPDGF-BB on tooth extraction socket healing

With respect to the effect of rhPDGF-BB for tooth extraction socket healing, there was only one primary outcome can be quantitatively analyzed, namely new bone formation rate. Three RCTs, Nevins 2011, Geurs 2014 and Ntounis 2015, compared the therapeutic impact of 0.3 mg/ml rhPDGF-BB on tooth extraction socket healing^28–30^. But because the data of Ntounis 2015 were derived from Geurs 2014 and did not include a measurement of the rate of new bone formation, we excluded it from the meta-analysis^29,30^. A 0.3 mg/ml concentration of rhPDGF-BB in the treatment group was associated with 2.16% more new bone formation rate than found in the control group. Though the difference goes in the predicted direction, the difference is not statistically significant (95%CI: −4.61, 8.93; p = 0.53). Furthermore, the heterogeneity of this study was low (χ^2^ = 0.89, I^2^ = 0%) (Fig. 4D).

### Systematic review of the utilizations of other growth factors in orofacial bone repair

For this section, we reviewed only those RCTs that used rhGFs as the intervention for orofacial bone regeneration. Other studies were excluded.

### Bone reconstruction for cleft lip and/or palate

#### Alveolar cleft repair

Ayoub 2016 applied rhBMP-7 to patients with alveolar cleft defects^31^. 11 patients received 3.5 mg of rhBMP-7 on a type I collagen carrier during surgery and were followed up for 6.6 years. Researchers measured the amount of bony infill via radiographic methods and assessed the effect of rhBMP-7 utilizing a Kindelan four-point scale. All unilateral alveolar cleft cases achieved scores at or above Grade II but in the two bilateral alveolar clefts, only one side experienced bone formation. Consequently, Ayoub 2016 encouraged a phase II trial to further evaluate the effect of rhBMP-7 for the remediation of alveolar defects^31^.

#### Maxillary sinus augmentation

Corinaldesi 2013 reported on the results of an RCT utilizing a parallel design^32^. The research studied the effect of rhBMP-7 on sinus floor augmentation. It dealt not only with clinical and radiological variables, but also with histological and histomorphometric factors. The results indicated that the usage of rhBMP-7 was actually associated with a lower rate of bone formation^32^. Van den Bergh 2000 carried out a pilot survey (which was not RCT)^33^. In this study three patients who received rhBMP-7 were compared with another three patients who received autogenous bone grafts. The data, however, were characterized by a quite significant heterogeneity of rhBMP-7 in different individuals. This heterogeneity led the authors to conclude that they were unable to make a reliable prediction as to whether the use rhBMP-7 would benefit maxillary sinus augmentation^33^.

The studies of Stavropoulos 2011 and Koch 2010 each reported on the application of rhGDF-5 to sinus augmentation. Their data, however, were from the same RCT^34,35^. They differed in that one of them evaluated the histological outcomes whereas the other dealt with clinical morphometric results. Both studies concluded that both the treatment groups that received rhGDF-5/β-TCP and the control groups that did not achieved similar levels of new bone formation and similar levels of bone quality^34,35^

#### Implant guided bone regeneration

Jung 2009 and Jung 2003 used 0.5 mg/ml rhBMP-2 to assess its effect on implant guided bone regeneration^36,37^. Both studies, however, are based on the same RCT, Jung 2009 being a simple extension of Jung 2003^36,37^. Both studies gave evidence that a combined use of xenogenic bone substitute mineral and rhBMP-2 can enhance implant-guided bone regeneration.

Santana2015 estimated the impact of 0.3 mg/ml rhPDGF-BB on implant-guided bone regeneration^38^. The results showed no significant difference along any measured parameter between the rhPDGF-BB group and the control group^38^.

## Disscusion

At present, the regeneration of lost orofacial bone continues to be a challenge requiring more effective solutions. Because of the limited efficacy of conventional therapies, patients with orofacial bone loss often suffer from a substantially lowered quality of life; their eating may be impaired, their speaking ability may be reduced, their appearance may be blemished and so on^1,39,40^. Improved therapeutic interventions are urgently required.

With the blossoming of research concerning the potential role of GFs in bone repair, rhGFs emerged as a promising candidate for orofacial bone regeneration. BMP-2, BMP-7, PDGF-BB and others have been widely used in the healing of bone. This provides a physiological rationale for considering their possible application to clinical situations. Besides, growing evidence from animal experiments and preliminary clinical trials have raised hopes that multiple autogenous GFs could possibly play positive roles in orofacial bone regeneration. Our previous study also provided strong evidence that 0.3 mg/ml rhPDGF-BB and 0.3% rhFGF-2 can effectively promote human periodontal bone repair^11^. In this study, we systematically reviewed RCTs dealing with the use of rhGFs in orofacial bone regeneration. We excluded studies dealing with periodontal diseases, due to the above-mentioned particularities of periodontal repair. We attempted to ascertain the clinical efficacy of rhGFs for orofacial bone regeneration in hopes of providing clinicians with more reliable guidelines as to the clinical use of rhGFs.

Our results indicate that rhBMP-2 is the most widely used rhGF in orofacial bone regeneration. Abundant *in vivo* and *in vitro* results indicate that BMP-2 can play a positive role in the chemotaxis, survival and early osteogenic differentiation of bone marrow mesenchymal stem cells (BMSCs). Researches utilizing multiple animal models also indicate that it can enhance the activities of osteoblasts. Therefore more than two decades ago, it began to be applied to the management of orofacial bone repair^7,41^. It has been used principally for sinus augmentation, tooth extraction socket healing, cleft alveolar reconstruction and implant-guided tissue regeneration. Our meta-analysis, however, indicates that the clinical efficacy of rhBMP-2 is far from satisfactory in sinus augmentation and CLP patients. These findings are at odds with that of most previous studies.

With respect to the utility of rhBMB-2 for sinus augmentation, our meta-analysis shows that it is not useful in the promotion of bone formation. But previous systematic reviews and meta-analysis have argued for the efficacy of rhBMP-2 in this regard. In our opinion, these earlier studies suffered from limitations which heavily weakened their conclusions. Firstly, their inclusion criteria are inconsistent or questionable. Jung *et al*. and Freitas *et al*., for example, inappropriately included two cohort studies as well as a case series report respectively in addition to RCTs^9,42^. Secondly, they fail to take into account certain sampling problems. Both Jung *et al*. and Mick *et al*., in their evaluation of the efficacy of rhBMP-2 on sinus augmentation included patients who received tooth extraction, thus ignoring the physiological and biological differences between tooth extraction socket healing and sinus augmentation. Thirdly, these studies suffered from a limited number of RCTs and from omission of relevant studies. In our analysis we have included five new RCTs and four new RCTs that had been updated, studies which were not included in the analyses of Jung *et al*. and Freitas *et al*. respectively. Rickert *et al*. suffered similar shortcomings with Jung *et al*. and Freitas *et al*.^9,42,43^. These three studies, namely Jung *et al*., Freitas *et al*., and Rickert *et al*., are systematic review without quantitative analysis. In 2015, Mick *et al*. conducted the first meta-analysis dealing with this aspect, but they failed to include two indispensable RCTs, Kim 2015, Froum 2014, as well as inappropriately included the data of Freitas 2013^24,27,44,45^. Specifically, the participants in Freitas 2013 were neither those who received tooth extraction nor those who need sinus augmentation^45^. Patients of this study had atrophic anterior maxilla therefore they needed alveolar ridge reconstruction before implant surgery. Taken together, Freitas 2013 should not be included in either the assessment of the effect of rhBMP-2 for sinus augmentation or the assessment for the healing of tooth extraction socket^45^. Besides, to avoid confounding variables, our study separated the results of tooth extraction healing from the estimation of sinus augmentation^9^. Jung *et al*. indicated that rhBMP-2 promoted sinus augmentation and that the effect is better as the dosage is increased^9^, but we document the questionable utility of rhBMP-2 for sinus augmentation no matter what concentration is used. (The two levels were 0.75 mg/ml and 1.5 mg/ml.)

Drawing lessons from these previous studies, we established a critical protocol to guide our literature search, systematically reviewed the literature, carefully distinguished the different populations and furthermore included more measurement parameters in our analysis. Our analysis indicates that rhBMP-2, far from facilitating sinus augmentation, may actually impair the restauration of bone height and bone width as well as the rate of new bone formation.

Some surprising negative findings about utilizing rhBMP-2 in CLP patients emerged in our research. More specifically: rhBMP-2 treatment groups seem to score lower than control groups on the parameters of bone formation rate and of increased bone volume. These results are different from those reported in the meta-analysis carried out in Wouter 2011, which argued for the positive impact that the use of rhBMP-2 among CLP patients could exert^10^. These contradictory findings may be due at least in part to the different studies that were included in the meta-analysis. We excluded from our research Herford 2007 because it was a retrospective controlled review rather than an RCT^10^. Wouter 2011 however included it. His conclusion regarding the positive effect of rhBMP-2 in this area may be due to the inclusion of a study that we explicitly excluded for the above-stated reasons. Interestingly, the results of the RCTs included in Wouter 2011 all documented the questionable role of rhBMP-2 in cleft bone reconstruction. In addition, for purposes of our meta-analysis we updated Canan Jr 2012 in an article that was published subsequently to Wouter 2011^17^. Another RCT (Neovius *et al*.) which was not included in either our work or Wouter 2011 concluded that the use of rhBMP-2 failed to produce better results in the regeneration of CLP patient’s cleft bones than conventional surgery^46^. Neovius *et al*. enrolled seven cleft children who randomly received 0.05 mg/ml rhBMP-2 or conventional autologous bone transplantation surgery. Disappointingly, six-months postoperative bone scans revealed that 0.05 mg/ml rhBMP-2 failed to induce almost any bone growth. A second surgery was subsequently performed on two patients utilizing an increased concentration of rhBMP-2 to 0.25 mg/ml. Six months after the second operation, CT results showed that the increased bone volume ratio produced by the heavier 0.25 mg/ml concentration of rhBMP-2 was weaker than that of a control group. (The differences were not statistically significant.) Even more problematic was the occurrence of severe gingival swelling during the first postoperative week in patients treated with the heavier rhBMP-2 dosage. Because the additional surgery procedures and the heavier concentrations of rhBMP-2 utilized in this study significantly differ from those used in the RCTs included in our study, this particular RCT was excluded^46^. But its findings give rise again to the issue of determining the most effective and safe concentration of rhBMP-2^47^.

According to Neovius *et al*. higher doses of rhBMP-2 are needed to achieve a positive therapeutic impact on CLP patients^46^. But there continues to be a longstanding debate about the effective and safe concentration of rhBMP-2 in human treatments. An experiment performed on rats involving femoral segmental defects cautioned that high BMP-2 concentration (0.15 mg/ml, 0.3 mg/ml and 0.6 mg/ml) resulted in inflammation and structurally abnormal bones^48^. Similar controversial issues have also arisen in the application of rhBMP-2 to other procedures in regenerative orthopedics^11,47^. In 2015 Katharina *et al*. systematically reviewed the literature and concludes that the effect of rhBMP-2 for bone healing is concentration-dependent; a lower concentration is more effective^47^. With respect to our results, further studies are necessary to determine if the currently available concentrations of rhBMP-2 are inappropriate (as appears to be the case) for sinus augmentation and CLP cleft treatment. More studies about the safety of this substance for use both on animals and humans are urgently called for.

But the possibility remains that changes in the concentration level or in the types of carriers may permit the use of rhBMP-2 for treatment of these two conditions. However, before exploring the effect of new concentrations or of new carriers of rhBMP-2 safety measures must be taken. It is important to determine whether changes in the concentrations or in the carriers utilized will indeed be safe for patients. In many fields of regenerative orthopedics, rhBMP-2 is believed to enhance new bone formation and to elevate the success of surgeries^2^. Our data, however, indicate that it is inappropriate to widely apply rhBMP-2 in the regeneration of orofacial bones and it is important for clinicians to follow suitable indications. Furthermore, there is a puzzling discrepancy between the positive results of the application of rhBMP-2 in animal experiments and the largely unsatisfactory results from the application of the same substance to humans in RCTs. The causal factors underlying this discrepancy deserve serious study.

Apart from rhBMP-2, our study indicates the possible efficacy of 0.3 mg/ml rhPDGF-BB in tooth extraction socket healing. The differences between treatment and control groups, however, have not yet been shown to be statistically significant. More RCTs are required to confirm what to date is simply a promising lead. But the optimistic result is consistent with our previous study, which confirmed the efficacy of 0.3 mg/ml rhPDGF-BB in periodontal regeneration. Whether it can prove effective for other functions, such as sinus augmentation or CLP cleft bone reconstruction, remains to be explored via more RCTs. The efficacy of other rhGFs, such as rhFGF-2, rhBMP-2, rhGDF-5, cannot yet be quantitatively evaluated because too few RCTs have been carried out regarding their impacts on orofacial bone repair. Adequate evidence-based-evaluations of their effect are currently unavailable.

Our data document the continuing challenges surrounding the widespread use of rhGFs in orofacial bone repair and bone regeneration. But our data indicate that no currently available concentrations of rhBMP-2 can be confidently recommended for the treatment of sinus augmentation or cleft alveolar issues. Further analysis is required of different concentrations and carriers used on well-selected patient samples before blindly recommending the use of rhGFs in orofacial bone regeneration. More experimental research on these issues is required to permit clinicians to make appropriate decisions.

## Supplementary information

Supplemetary data 1

## Data Availability

All data generated or analyzed during this study are included in this published article and its Supplementary Information files.
