# Supplementary material for: Efficacy of Recombinant Human BMP2 and PDGF-BB in Orofacial Bone Regeneration: A Systematic Review and Meta-analysis
Source: Sci Rep. 2019 May 30;9:8073. doi: 10.1038/s41598-019-44368-z (PMC6542831; doi:10.1038/s41598-019-44368-z)
Supplement: Supplementary file 1 — Supplemetary data 1 [file 41598_2019_44368_MOESM1_ESM.pdf]

Efficacy of Recombinant Human BMP2 and PDGF-BB in Orofacial Bone Regeneration: A Systematic Review and Meta-analysis

Feifei Li[1, 3, #], Fanyuan Yu[1, 2, #], Xueyang Liao[1, 3], Chenzhou Wu[1, 4], Yitian Wang[1], Chunjie Li[1, 4], Feng Lou[1, 2], Boer Li[1, 2], Bei Yin[1, 2], Chenglin Wang[1, 2], Ling Ye[1, 2*]

1 State Key Laboratory of Oral Diseases & National Clinical Research Center for Oral Diseases, West China Hospital of Stomatology, Sichuan University
2 Department of Endodontics, West China Stomatology Hospital, Sichuan University
3 Department of Pediatric Dentistry, West China Hospital of Stomatology, Sichuan University
4 Department of Oral Maxillofacial Surgery, West China Hospital of Stomatology, Sichuan University
* Correspondence may be addressed to yeling@scu.edu.cn
# These authors contributed equally to this work.

S1

PRISMA 2009 Flow Diagram

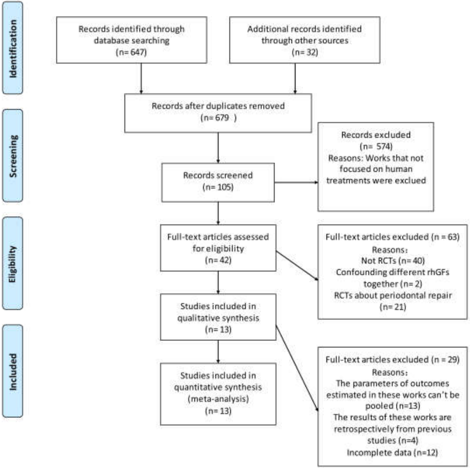

From: Moher D, Liberati A, Tetzlaff J, Altman DG, The PRISMA Group (2009). *Preferred Reporting Items for Systematic Reviews and Meta-Analyses*: The PRISMA Statement. PLoS Med 6(7): e1000097. doi:10.1371/journal.pmed1000097

S1: Study selection.

For more information, visit www.prisma-statement.org.

S2

A

Study or Subgroup	rhBMP-2 Mean	rhBMP-2 SD	rhBMP-2 Total	control Mean	control SD	control Total	Weight	Mean Difference IV, Random, 95% CI	Risk of Bias A B C D E F G
Froum 2014	93.2	67.44130074	24	4.6	100.9929	12	52.1%	88.60 [25.41, 151.79]	? ? + + ? ? ?
Philip 2005	137	77	15	350	243	13	47.9%	-213.00 [-350.72, -75.28]	? ? ? + + + ?
**Total (95% CI)**			**39**			**25**	**100.0%**	**-55.74 [-351.03, 239.55]**	

Heterogeneity: Tau² = 42492.79; Chi² = 15.22, df = 1 (P < 0.0001); I² = 93%
Test for overall effect: Z = 0.37 (P = 0.71)

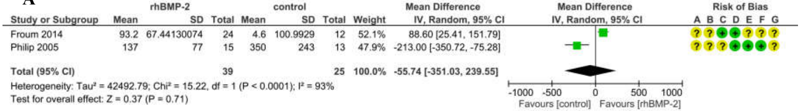

B

Study or Subgroup	rhBMP-2 Mean	rhBMP-2 SD	rhBMP-2 Total	control Mean	control SD	control Total	Weight	Mean Difference IV, Random, 95% CI	Risk of Bias A B C D E F G
Froum 2014	93.2	67.44130074	24	4.6	100.9929	12	51.7%	88.60 [25.41, 151.79]	? ? + + ? ? ?
Philip 2005	108.090909	68.11954732	33	350	243	13	48.3%	-241.91 [-376.03, -107.79]	? ? ? + + + ?
**Total (95% CI)**			**57**			**25**	**100.0%**	**-71.14 [-394.86, 252.57]**	

Heterogeneity: Tau² = 51756.97; Chi² = 19.09, df = 1 (P < 0.0001); I² = 95%
Test for overall effect: Z = 0.43 (P = 0.67)

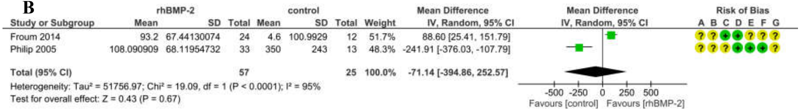

S2:Forest plot of comparison: the effect of rhBMP-2 for sinus augmentation; outcome: new bone density.
(A) The assessment of 1.5mg/ml rhBMP-2 for the regulation of new bone density. (B) The assessment about the concentrations of 0.75mg/ml and 1.5mg/ml of rhBMP-2 for the regulation of new bone density.
